# The Effect of Adding Neuromuscular Electrical Stimulation with Endurance and Resistance Training on Exercise Capacity and Balance in Patients with Chronic Obstructive Pulmonary Disease: A Randomized Controlled Trial

**DOI:** 10.1155/2020/9826084

**Published:** 2020-09-29

**Authors:** Amal Acheche, Marwa Mekki, Thierry Paillard, Zouhair Tabka, Yassine Trabelsi

**Affiliations:** ^1^Laboratoire de Recherche Physiologie de l'Exercice et Physiopathologie: de l'intégré au moléculaire « Biologie, Médecine et Santé » (LR19ES09), Faculté de Médecine de Sousse, Sousse 4002, Tunisia; ^2^Biology Department, Faculty of Sciences of Sfax, Sfax3038, Tunisia; ^3^Movement, Balance, Performance and Health Laboratory (EA 4445), University of Pau and des Pays de l'Adour, Pau 64012, France

## Abstract

This study investigated the effectiveness of adding neuromuscular electrical stimulation (NMES) to endurance training (ET) and resistance training (RT) on exercise tolerance and balance in COPD patients. 42 patients were assigned randomly to the ET + RT + NMES group (*n* = 22) or ET + RT group (*n* = 20). Two training programs were performed including 72 sessions. The center of pressure (CoP) displacement in the mediolateral direction (CoPML), in the anteroposterior direction (CoPAP), and the center of pressure velocity (CoPV) were recorded using a stabilometric platform with eyes open (EO) and eyes closed (EC). Time up and go and Berg Balance Scale tests, 6-minute walking test (6MWT), and the maximal voluntary contraction (MVC) were measured before and after the intervention. The walking distance, the dyspnea, and the heart rate were improved after the training period (*p* < 0.001) for both groups (*p* < 0.05). The ET + RT + NMES group showed better improvement than the ET + RT group in terms of 6MWD. CoP_ML_, CoP_AP_, and CoP_V_ were significantly (*p* < 0.001; *p* < 0.05; *p* < 0.001, respectively) more improved in EO and EC conditions in the ET + RT + NMES group than the ET + RT group. BBS, TUG, and MVC values improved in both groups after the training (*p* < 0.001). The performances in TUG and MVC tests were significantly greater in the ET + RT + NMES group than those in the ET + RT group (*p* < 0.01; *p* < 0.001, respectively). Combining NMES, RT, and ET improves balance in patients with COPD.

## 1. Introduction

Chronic obstructive pulmonary disease (COPD) patients have demonstrated prominent balance impairment [1] compared to healthy age-matched controls [2]. The deficit in balance control is identified as one of the risk factors of falls in subjects with COPD and alters their daily-life activities [3, 4]. Muscle weaknesses, physical inactivity, and limited mobility are well known to weaken balance control [2–4]. Indeed, the reduced quadriceps' muscle strength is associated to balance impairment in patients with COPD [5].

Exercise training, as a pillar of pulmonary rehabilitation, plays an essential role in improving muscle strength, exercise tolerance, and quality of life [6].

Despite the well-known positive effects of exercise training in patients with COPD, the best modality of exercise is still under discussion, and it depends on the physiologic requirement. In fact, various training modalities were used seeking additional benefits. Endurance training (ET) aimed to improve cardiorespiratory fitness, increase physical activity, and decrease dyspnea and fatigue [7]. Resistance training is an exercise modality in which local muscle groups are trained by repetitive lifting of relatively heavy loads [7]. It appears to be a good option for those with lower-limb muscle dysfunction. It limits the muscle strength impairment and promotes the muscle strength and force [7]. The addition of RT to ET showed that it confers more beneficial effects [7].

Neuromuscular electrical stimulation (NMES), an emerging alternative, seems to be suitable for patients who are unable to exercise [8]. NMES has gained attention due to its wide application. For the COPD patients, NMES induces several benefits such as gain in muscle strength, increased exercise tolerance, and better quality of life [9, 10].

Recently, some studies focused on improving the balance and reducing the risk of falls [11, 12]. Beauchamp and coworkers demonstrated the effectiveness of balance training as part of PR for improving balance performance, muscle strength, and self-reported physical function in patients with COPD [12]. Mekki et al. showed that the combination of ET and NMES focusing on postural muscles could improve balance impairment [13]^.^

Based on these research studies, we hypothesize that the combination of NMES, RT, and ET would improve balance, as well as the exercise tolerance in patients with COPD. We hypothesize that combined training (ET + RT + NMES) induces additional physiological adaptations than ET alone or RT alone or NMES alone.

The aim of our study was to compare the effect of adding NMES to RT and ET on exercise tolerance and balance in patients with COPD.

## 2. Materials and Methods

### 2.1. Study Population

Forty-two male patients accepted to participate in the study. They were recruited from the department of physiology and lung function testing of Farhat Hached Hospital in Sousse in the period between 15/01/2016 and 15/09/2016. The criteria of inclusion were COPD diagnosed by pulmonary function testing, clinically stable, without other obstructive diseases, absence of heart diseases, absence of neuromuscular diseases, and fall in the past five years or recent near-fall. Individuals were excluded if they presented severe psychiatric, neurologic, or musculoskeletal conditions and/or instable cardiovascular diseases.

**Figure 1:**
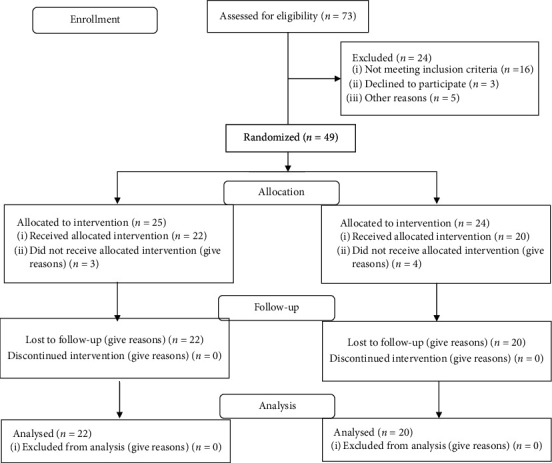
Flow diagram of subjects' recruitment.

The patients were assigned randomly to the ET + RT + NMES group (*n* = 22) or ET + RT group (*n* = 20) using a computer-generated randomization list **(**Figure 1, flowchart). The study (clinical trial number: NCT03577080**)** was approved by a research ethics committee of Farhat Hached Hospital of Sousse. The protocol and the objectives of the study were explained to patients.

### 2.2. Measurements

The patients were evaluated at the baseline and after 24 weeks of training (3 times a week for 90 minutes per session). On the first day, after obtaining written informed consent, measurements of pulmonary function at rest, weight, and height were done. On the following day, the subjects performed the six-minute walking test (6MWT). Spirometry, balance assessment, and maximal voluntary contraction (MVC) of the muscle quadriceps were also recorded.

#### 2.2.1. Pulmonary Function Testing

All patients underwent pulmonary function testing with the measure of forced expiratory volume in 1 s (FEV_1_) and forced vital capacity (FVC). Testing was performed using Spirometry Zan 100 (Inspire Health GmbH, Germany) according to the European Respiratory Society recommendations [14].

#### 2.2.2. Exercise Tolerance

The 6MWT evaluates the functional exercise capacity. The patient was invited to walk as far as possible along a flat corridor [15]. Tests were conducted according to the European Respiratory Society/American Thoracic Society technical standard [16]. Dyspnea and breathlessness were measured using the Borg scale. The heart rate (HR) and oxygen saturation (SO_2_) were recorded as well during the test by a portable Spiropalm (COSMED, Rome, Italy). At the end of the test, the six-minute walking distance (6MWD) was recorded.

#### 2.2.3. Force Assessment

Patients began with a warm-up phase of the leg extensor muscle consisting of cycling for 5 min. Next, they performed three maximal voluntary contractions (MVC) separated by 5 min of rest to estimate the maximum voluntary force level (Globus Ergo system, TESYS 1000, Italy). Patients were required to sit with 90°hip flexion and 90°knee flexion. Stabilization straps were positioned across the chest, and the arms were crossed upon the chest.

#### 2.2.4. One-Repetition Maximum (1-RM)

The 1-RM test (kg) is used for assessing muscle strength. It is defined as the maximal weight that can be lifted once with the correct lifting technique (quadriceps, gluteus, hamstrings, and calf muscles).

#### 2.2.5. Dynamic Balance Assessment

*(1) Berg Balance Scale (BBS)*. The fourteen-item BBS was chosen as the primary outcome because it is the most accepted measure of balance for elderly people. The test evaluation was graded from 0 (unsure/unsafe) to 4 (independent/safe) with a maximum score of 56. The measurement derived using the BBS has shown internal consistency, intra-, and inter-rater reliability for determining fall risk in older adults [17].

*(2) Time Up and Go (TUG)*. TUG was a simple test. The patient was initially sitting on a standard armchair, got up, walked three meters, with a comfortable pace, turned, walked back, and sat down again. A practice test was performed without being recorded. TUG had high intra- and intertester reliability and predictive validity for falls in community-living adults [18, 19].

*(3) Static Balance Assessment*. Three tests were performed using a stabilometric platform. The subject was asked to stand, as still as possible, 25.6 seconds on the platform (PostureWin, Techno Concept, Cereste, France) barefoot, with arms along his body, eyes open (EO), and then eyes closed (EC) [20]. The center of pressure (CoP) displacement in the mediolateral direction (CoPML), in the anteroposterior direction (CoPAP), and the center of pressure velocity (CoPV) were recorded using a stabilometric platform with eyes open (EO) and eyes closed (EC).

*(4) Intervention*. The RT + ET group received 45 minutes of endurance training (ET) combined with 40 minutes of resistance training (RT). The intervention group (the RT + ET + NMES group) received ET program (45 min) + NMES (20 min) + RT (20 min).

### 2.3. Endurance Training (ET)

Either group benefited three times a week for 24 weeks (72 sessions). The training session lasted 90 minutes. Each subject had to wear a pulse oximeter to control the saturation (SO_2_) and the heart rate (HR) during the training session.

A 5-minute warm-up, including exercises of stretching, was performed. Both groups received an individualized endurance training program which consisted of walking on the Ergo cycle for 45 minutes at 60–70% of the maximum HR reached at the end of 6MWT.

The training emphasized interval-type exercise; periods of high-intensity exercise were alternated with active recovery (10 min at 60–70% of the maximum HR alternated with 5 min of active recovery repeated over 45 min).

### 2.4. Resistance Training (RT)

Both groups performed exercises for upper and lower limbs, two sets of 8–10 repetitions, with 1 minute break between exercises and two minutes between sets.

The RT is targeted to strengthen the lower and the upper limb muscles (biceps, triceps, deltoids, and pectoral muscles). The exercises consisted of leg extension, leg press, and leg curl. The training load of lower limb muscles started at 50% 1RM; then, it was set to increase to 80% 1RM. The load was set in kilograms (kg).

### 2.5. Neuromuscular Electrical Stimulation (NMES)

The NMES exercise consisted in completing three sessions a week for 24 weeks. During the NMES session, the subject was seated, and the foot was free with 60°leg flexion for maximal contraction to be produced. Quadriceps and calf muscles of both legs were stimulated electrically with two electrical stimulators (Globus 400) with four output channels. Sixteen self-adhesive (in both legs), rectangular, bipolar adhesive electrodes (5 × 5 cm^2^) were applied, and each of them was located on one of the anatomical motor points of the quadriceps and calf. The target muscles were vastus medialis, rectus femoris, and vastus lateralis muscles. Four electrodes were applied to the semitendinosus, and semimembranosus muscles' motor points were previously determined, and the other electrodes were placed on the soleus and gastrocnemii (over both medial and lateral gastrocnemii) muscle motor points. This training was assessed with the following parameters: (1) symmetrical biphasic pulses fixed at 50 Hz frequency, (2) 400 *μ*s pulses, (3) intensity amplitude 15–20 mA, and (4) 10/30 s duty cycle [9]. These parameters were selected to minimize skin irritation and muscular fatigue.

#### 2.5.1. Statistical Analysis

Statistical analyses were realized using Statistica for Windows software (version 6.0, StatSoft, Inc., Tulsa). The normality was tested and determined using the Kolmogorov–Smirnov test.

A two-way analysis of variance (ANOVA) was used to compare baseline characteristics (anthropometric and pulmonary function) (2 groups (ET + RT and ET + NMES + RT) × 2 periods (before training and after training)).

6MWT parameters, MVC, and dynamic balance parameters (BBS and TUG) were analyzed by means of two-way ANOVA.

Static balance parameters (COP) were analyzed using a repeated-measure analysis of variance (ANOVA) with three factors: group (ET + RT and ET + RT + NMES), period (before and after), and vision (EO and EC). A post hoc test (Tukey) was performed to further analyze these results in detail. The level of significance was set at *p* < 0.05.

## 3. Results

### 3.1. Pulmonary Function Testing

The anthropometric characteristics and pulmonary function parameters of patients with COPD are provided in Table 1. No significant differences were mentioned among the two groups before and after the training program.

All statistical results of ANOVA analysis of 6MWT (6MWTD, SpO2 rest and peak, dyspnea rest and peak, and HR rest and peak), dynamic balance outcomes (TUG and BBS), MVC, and static balance (CoP_ML_, CoP_AP_, and CoP_V_) were recapitulated in Tables 2 and 3.

### 3.2. Exercise Tolerance

The post hoc test (Table 4) showed that the 6MWD, the SpO2 peak, and the MVC enhanced for both groups (*p* < 0.001) after the intervention. The 6MWD and the MVC increased significantly (*p* < 0.001) more for the ET + RT + NMES group than for the ET + RT group after the training program. Dyspnea peak and HR (at rest) dropped significantly after the training program (*p* < 0.001) in both groups.

### 3.3. Balance Outcomes

The post hoc test (Table 5) showed that the training program decreased CoP_ML_ in both groups (*p* < 0.001) and conditions (*p* < 0.001). CoP_ML_ decreased significantly in both conditions for the ET + RT + NMES group compared to the control (*p* < 0.001).

The post hoc test demonstrated a significant decrease in CoP_AP_ for the ET + RT + NMES group (*p* < 0.001) in both conditions and for the ET + RT group in OE (*p* < 0.05) and CE (*p* < 0.001) conditions. CoP_AP_ decreased more in both conditions (*p* < 0.05) for the ET + RT + NMES group compared to the ET + RT group. CoP_V_ decreased (*p* < 0.001) in both visual conditions after both training programs. CoP_V_ decreased more for the ET + RT + NMES group in EO (*p* < 0.001) and EC (*p* < 0.05) conditions than for the ET + RT group.

Concerning dynamic balance outcomes (Table 5), the post hoc test revealed that TUG scores dropped significantly after the training programs (*p* < 0.001) in both groups. Better improvement was obtained in the ET + RT + NMES group (*p* < 0.01) compared to the ET + RT group. The BBS scores increased significantly after the training programs (*p* < 0.001) in both groups.

## 4. Discussion

Our study aimed to compare two combined training modalities (RT + ET + NMES vs. RT + ET) on exercise tolerance and balance in patients with COPD.

The main finding suggests that combined training, including NMES, improved better the static and dynamic balance and exercise tolerance, as well as the lower limb strength, compared to training without NMES in patients with COPD.

As expected, the pulmonary characteristics have not changed after six months of training. These results are in agreement with previous studies [21].

The 6MWD increased significantly after the training program in favor of the ET + RT + NMES group. This could be explained by improved physiological conditions, a good exercise response, and a reduction of dyspnea [21]. The improved walking distance may also be related mostly to the enhanced muscle strength. The reduction of ventilatory requirements might also contribute to the enhancement of 6MWD distance [8]. Reducing the dyspnea might be the result of a better utilization of oxygen by the exercising muscles and, thus, ventilator needs for a given work rate [22]. The heart rate was decreased, as well, in rest and during exercise. This decrease could indicate an improvement of the cardiovascular condition of the patients [23]. Besides, a decline of alveolar hypoxemia could have a positive impact on cardiovascular function [24, 25]. The decrease in the heart rate still supported the finding of physiological benefits of exercise training [23]. Our finding supports those of previous studies that showed that NMES alone or combined with a pulmonary rehabilitation on severe COPD patients had reduced the dyspnea, exercise capacity, and femoris quadriceps strength in subjects with COPD [8, 21].

Improvement in leg muscle strength in the ET + RT + NMES group could be explained by increasing capillarization and fiber-type plasticity [26]. Besides, another explanation could be taken into consideration that skeletal muscle hypertrophy is achieved by positive protein balance and fusion of satellite cells to myofibers after exercise training in COPD patients [26]. Similar to what was found in previous studies, leg muscle strength has improved significantly more in the ET + RT + NMES group than in the ET + RT group [21]. Our results are in agreement with Bezzera and coworkers, who found a significant increase in muscle strength of knee extensors and flexors when applying combined training (NMES + ET) in the elderly [27].

The major finding of the current study is the improvement of static (CoP_ML_, CoP_AP_, and CoP_V_) and dynamic (TUG and BBS) balance outcomes for the ET + RT + NMES group compared to the ET + RT group. These results could be explained by an increase of muscle strength [28]. In fact, it is well documented that poor balance in COPD subjects is associated with muscle weakness, so the improvement of muscle strength will have a positive impact on restoring balance deficits [29], especially in frail subjects [30].

Besides, NMES would improve the excitability and recruitment of muscle fibers and result in higher force and better coordination in the elderly [31]. Furthermore, NMES could lead to neural plasticity and improve voluntary muscle activation and consequently increase muscle strength [32]. Additionally, the muscle strength expressions related not only to the activation of postural muscles (facilitated by the stretch reflex, reciprocal innervation, myotendinous and joint stiffness) but also to the inhibition of their antagonists (disfacilitated by the tendinous reflex) through postural neural networks [33].

The balance impairment is more critical in the medial-lateral (CoP_ML_) direction in rest and during movements [1]. Hence, the displacement in the mediolateral (CoP_ML_) and anteroposterior (CoP_AP_) improved more in the ET + RT + NMES group than the ET + RT group. Some reasons could explain the enhancement of displacement in the medial-lateral direction: first, the training targeted strength and endurance of hip and trunk muscles, which could improve their contribution to the displacement in the medial-lateral balance control [34, 35]. Second, a reduction of dyspnea and breathlessness might lead to a better contribution of hip and trunk mediolateral balance [36]. Third, an increased physical activity might also enhance the sensory function of the body movement and position for interpretation and response to the movement of the center of pressure [37].

CoP improvement might be due to the improvement of the somatosensory function of the lower limbs by adding NMES. The exercise training could enhance the patients' ability to integrate the somatosensory and vestibular inputs, becoming less reliant on the visual input while applying appropriate sensory strategies to control their posture and prevent falls [37].

Moreover, NMES proved its efficacy by activation of large and small motor units (MUs) [38]. NMES leads to their activation, imposing a contractile activity of both MU even relatively low intensities [39]. These facts might explain that a superficial muscle such as the rectus femoris is more highly activated in both NMES groups than the ET + RT group [40].

Studies conducted in different diseases support our results. Amiridis et al. chose the ankle dorsiflexors to benefit from NMES combined with physical training. They reported that combined training reduced postural sway and improved the balance control in the anterior-posterior direction compared to the medial-lateral axis [28]. Dos Santos et al. showed in a review that combined training with NMES improved muscle balance (quadriceps and hips) in people with patellofemoral dysfunction [41]. In COPD, Mekki et al. found an improvement in postural outcomes after adding the NMES to PR [13].

Concerning dynamic balance outcomes, a significant improvement was identified. BBS scores increased significantly in both groups. A better improvement in TUG scores was detected in the ET + RT + NMES group. Cho et al. showed that four weeks of treadmill training combined with NMES onto the gluteus medius and tibialis anterior muscles enhanced the BBS scores significantly. Thus, this combined training seems to be effective in improving balance performances, gait velocity, cadence, single support time, temporal asymmetry, and spatial asymmetry in stroke patients with chronic hemiparesis [42]. You et al. showed that NMES combined with standard rehabilitation in patients with stroke affected mobility of lower limbs and balance performances and improved BBS scores positively [43].

The main limitation of the study was the reduced number of patients. Given the heterogeneity of the response to NMES in the literature, emerging evidence suggested responders and nonresponder patients to NMES. Another limitation should be taken for consideration: the cardiopulmonary testing technique. The cardiopulmonary function could be evaluated by other techniques such as cardiopulmonary exercise testing and could give more measurements.

## 5. Conclusions

The combination of ET, RT, and NMES improves static and dynamic balance likewise in exercise tolerance in patients with COPD. This training program decreased the balance impairment and reduced dyspnea and fatigue. It could reduce the risk of falls and can be part of pulmonary rehabilitation for the patients with COPD.

## Figures and Tables

**Table 1:** Anthropometric characteristics and pulmonary function before and after the intervention.

Patient characteristics	Interventional group (*n* = 22)	Control group (*n* = 20)	*p* value
Gender (M/F)	22/0	20/0	–
Age (years)	63 ± 4	62 ± 6	0.53
Height (cm)	166 ± 9	168 ± 7	0.46
Weight (kg)	67 ± 15	67 ± 13	0.92
Body mass index, BMI (kg/m^2^)	24.09 ± 5.97	25.14 ± 5.68	0.80
Current smoker/exsmoker	0/22	0/20	–
Pack/years	47 ± 6	49 ± 5	0.20
Dyspnea (Borg scale)	1.64 ± 0.72	1.60 ± 0.59	0.86
FEV_1_ (l) Baseline Follow-up	1.69 ± 0.53 1.66 ± 0.60	1.73 ± 0.46 1.73 ± 0.47	0.78
FEV_1_ (%) Baseline Follow-up	54.13 ± 19.85 54.10 ± 20.90	54.80 ± 15.74 54.70 ± 18.12	0.92 0.89
FVC (l) Baseline Follow-up	2.95 ± 0.78 2.69 ± 0.61	3.16 ± 0.36 3.14 ± 0.33	0.29 032
FVC (l) Baseline Follow-up	76.31 ± 12.77 79.19 ± 13.16	80.1 ± 10.53 75.25 ± 10.49	0.30 0.71
FEV_1_/FVC (%) Baseline Follow-up	57.12 ± 14.30 61.18 ± 15.07	54.80 ± 6.69 55.12 ± 6.61	0.11 0.13

Data are represented as mean ± standard deviation units. BW: body weight; BMI: body mass index; FEV_1_: forced expiratory volume in one second; FVC: forced vital capacity.

**Table 2:** Statistical results of the ANOVA analysis of exercise tolerance, dynamic balance, and maximal voluntary contraction parameters (2-way ANOVA).

Parameters	2-way ANOVA					
	Group		Period		Group × period	
	F-value	*p*	F-value	*p*	F-value	*p*
6MWTD (m)	5.83	<0.01	173.49	<0.001	5.95	0.024
SpO2 peak	0.1	0.81	297.9	<0.001	6.7	0.017
Dyspnea peak	0.56	0.46	294.74	<0.001	3.08	0.09
HR rest	1.40	0.25	139.98	<0.001	0.25	0.62
HR peak	0.03	0.86	16.42	0.0006	0.31	0.58
TUG (s)	4.82	0.04	399.68	<0.001	4.72	0.04
BBS (score)	0.91	0.1	305.60	<0.001	13.07	0.002
MVC (N)	4.10	0.05	431.61	<0.001	9.97	0.005

6MWTD: six-minute walking test distance; SpO_2_: peripheral oxygen saturation; HR: heart rate; TUG: time up and go test; BBS: Berg Balance Scale; MVC: maximal voluntary contraction.

**Table 3:** Statistical results of the ANOVA analysis of static balance parameters (3-way ANOVA).

Parameters	3-way ANOVA					
	CoP_ML_ (mm)		CoP_AP_ (mm)		CoP_v_ (mm/s)	
	F-value	*p*	F-value	*p*	F-value	*p*
Group	7.49	0.01	4.25	0.05	19.19	<0.001
Period	458.23	<0.001	139.4	<0.001	619.91	<0.001
Vision	119.42	<0.001	500.02	<0.001	282.31	<0.001
Group × period	31.59	<0.001	8.85	<0.01	19.48	<0.001
Group × vision	0.70	0.41	0.00	0.98	4.08	0.05
Period × vision	9.17	<0.001	7.46	<0.01	22.38	<0.001
Group × period × vision	0.09	0.76	0.08	0.77	0.055	0.81

CoP_ML_: center of the pressure in the mediolateral direction; CoP_AP_: center of the pressure in the anterior-posterior direction; CoP_v_: oscillation velocity of the pressure center.

**Table 4:** 6MWT parameters and maximal voluntary contraction before and after the intervention.

Parameters	Intervention group (*n* = 22)	Control group (*n* = 20)	*p* value
6MWD Baseline Follow-up	418 ± 105 542 ±136	427 ± 61 523 ± 68	**0.28** **<** **0.05**
%SpO2 rest Baseline Follow-up	93 ± 2 94 ± 2	93 ± 1 94± 1	**0.72** **0.46**
%SpO2 peak Baseline Follow-up	88 ± 2 91± 1	88 ± 1 90± 1	**0.63** **0.17**
Dyspnea rest (Borg) Baseline Follow-up	1.64 ± 0.72 0.91 ± 0.61	1.60 ± 0.59 0.95 ± 0.45	**0.86** **0.37**
Dyspnea peak (Borg) Baseline Follow-up	4.18 ± 1.44 2.59 ± 0.96	4.25 ± 0.85 3.10 ± 0.79	**0.85** **0.06**
HR rest (bpm) Baseline Follow-up	82 ± 7 80± 6	84 ± 6 81± 5	**0.41** **0.53**
HR peak (bpm) Baseline Follow-up	118 ± 13 112±10	118 ± 8 113±5	**0.91** **0.56**
MVC (Newton meter) Baseline Follow-up	356 ± 31 440± 25	355 ± 30 413± 26	**0.95** **<** **0.001**

ET: endurance training; RT: resistance training; NMES: neuromuscular electrical stimulation; MVC: maximal voluntary contraction; SpO_2_: peripheral oxygen saturation; Borg: modified Borg dyspnea scale; HR: heart rate; bpm: beats per minute.

**Table 5:** Mean values and standard deviations of static and dynamic balance outcomes before and after the intervention.

	Intervention group: ET + RT + NMES				Control group: ET + RT			
	Before		After		Before		After	
	EO	EC	EO	EC	EO	EC	EO	EC
CoPML (mm)	118.01 ± 13.77	146.69 ± 13.93	84.54 ± 10.22 ^*∗∗∗*^*EEE*	103.67 ± 6.53 ^*∗∗∗*^*EEE*	118.36 ± 16.96	144.01 ± 10.96	99.30 ± 12.76 ^*∗∗∗*^	116.78 ± 5.89 ^*∗∗∗*^
CoPAP (mm)	175.03 ± 10.11	247.34 ± 14.72	160.49 ± 8.24 ^*∗∗∗*^*E*	224.16 ± 17.18 ^*∗∗∗*^*E*	178.16 ± 10.41	249.58 ± 19.59	169.55 ± 12.62 ^*∗*^	233.64 ± 14.42 ^*∗∗∗*^
CoPV (mm/s)	10.03 ± 1.35	13.95 ± 0.96	7.48 ± 1.46 ^*∗∗∗*^*EEE*	10.47 ± 0.91 ^*∗∗∗*^*E*	10.83 ± 1.15	14.02 ± 1.27	9.40 ± 0.32 ^*∗∗∗*^	11.40 ± 0.82 ^*∗∗∗*^
TUG (s)	13.57 ± 0.71		10.06 ± 0.48 ^*∗∗∗*^*EE*		13.75 ± 0.98		10.71 ± 0.70 ^*∗∗∗*^	
BBS (score)	47.77 ± 4.49		53.68 ± 1.78 ^*∗∗∗*^		45.70 ± 1.79		55.10 ± 1.07 ^*∗∗∗*^	

ET: endurance training; RT: resistance training; NMES: neuromuscular electrical stimulation; OE: open eyes; CE: closed eyes; CoP_ML_: center of the pressure in the mediolateral direction; CoP_AP_: center of the pressure in the anterior-posterior direction; CoP_v_: oscillation velocity of the pressure center; TUG: time up and go; BBS: Berg Balance Scale. ^*∗*^Significant (*p* < 0.05) in the same group in the same visual condition before and after the intervention. ^*∗∗*^Significant (*p* < 0.01) in the same group in the same visual condition before and after the intervention. ^*∗∗∗*^significant (*p* < 0.001) in the same group in the same visual condition before and after the intervention. *E*: significant (*p* < 0.05) between groups in the same visual condition. *EE*: significant (*p* < 0.01) between groups in the same visual condition. *EEE*: significant (*p* < 0.001) between groups in the same visual condition. Variables are expressed as mean ± standard deviation units.

## Data Availability

The data used to support the findings of this study are included within the article.
